# Means of Escape in Case of Fire

**Published:** 1910-01-15

**Authors:** 


### FIRES IN HOSPITALS.

### MEANS OF ESCAPE IN CASE OF FIRE.

An Architect's Protest against the London County Council Regulations.

Much attention has been given of late years to the question of providing adequate means of escape from the wards and other parts of hospitals in case of an outbreak of fire, and much money has been spent in the construction of outside staircases and other appliances with that object. The subject is an important one, and should be considered with the utmost care. On the other hand, it is most necessary to avoid anything in the shape of panic or to incur expenditure in works of little or no practical value. In the first place it is necessary to differentiate between buildings constructed with wooden floors and staircases and those mainly built of fire-resisting materials. The planning of the several parts of a hospital is also an element of first importance. For example, a hospital built on the pavilion system, with its wards in isolated blocks connected with each other and with the main building by one story corridors is clearly better arranged for checking the spread of fire than one in which wards, official quarters, stores, and kitchen offices are jumbled together in one continuous pile. Again it is surely reasonable when considering the question of escape in case of fire to consider first what the reasonable chances are of a fire breaking out at all. To take a concrete example, we will, without specifying the particular institution, describe briefly the arrangements of a modern provincial hospital for some 200 beds. In the centre is the front administration block, with the official offices and residences for staff and servants. Connected to this by a one-story corridor with a flat roof of steel and concrete is a block in which are placed the kitchen offices and stores and the staff mess rooms. Between these two blocks the main corridor, a one-story erection with a wood and glass roof, runs right and left, and gives access to the ward pavilions, which project at right angles on each side. One staircase serves each pair of pavilions, and an intervening lobby cuts the wards and their offices off from the corridor and staircase on each floor. These ward pavilions contain nothing whatever but the wards and their offices. The walls are of brick and the floors of steel joists and concrete, and the surface of the floors is finished with marble terrazzo. It is not too much to say that nothing short of incendiarism could cause an outbreak of fire in any of these pavilions, and should an outbreak occur in either of the administration blocks it is physically impossible that it could spread to either of the ward blocks. In these circumstances what justification is there for requiring an outside escape staircase to each of these pavilions? Surely none; but if the hospital were in London instead of being in a country town such a requirement would most certainly be made by the London County Council.

Further, it may be asked what practical good would such a staircase serve in case of a panic? A very large proportion of the patients would be quite incapable of using the staircase themselves, and to carry all the helpless patients down an iron staircase of the ordinary kind would be an impossibility.

It must not be forgotten that panic is quite as dangerous in a way as an actual fire, and must be dealt with as cautiously. In case, therefore, of an alarm of fire being started in a ward block means must be at hand for removing the patients quickly and safely. Most modern hospitals are now provided with balconies leading out of the wards, and if these are sufficiently spacious all the patients could quickly and without difficulty be taken or carried out, and would then be in perfect safety. Balconies are in these days a necessity; but outside staircases are not, and, moreover, are costly to erect and to maintain.

With buildings of the older class, whose arrangement is of an obsolete incoherent type and whose construction cannot be classed as fire-resisting, the escape staircase becomes a necessity; but it should be supplemented by broad landings or balconies on to which helpless patients can be wheeled or carried out of harm's way.

